# Lineage plasticity and histological transformation: tumor histology as a spectrum

**DOI:** 10.1038/s41422-025-01180-x

**Published:** 2025-09-30

**Authors:** Xiaoling Li, Eric E. Gardner, Sonia Molina-Pinelo, Clare Wilhelm, Ping Mu, Álvaro Quintanal-Villalonga

**Affiliations:** 1 Department of Urology, Yale School of Medicine, Yale University, New Haven, CT USA; 2 Yale Cancer Center, Yale University, New Haven, CT USA; 3 Meyer Cancer Center, Weill Cornell Medicine, New York, NY USA; 4 Institute of Biomedicine of Seville (IBiS), HUVR, CSIC, Universidad de Sevilla, Seville, Spain; 5 Department of Medicine, Thoracic Oncology Service, Memorial Sloan Kettering Cancer Center, New York, NY USA

**Keywords:** Tumour heterogeneity, Cancer therapy

## Abstract

Lineage plasticity, the ability of cells to transition to an alternative phenotype as a means for adaptation, is an increasingly recognized mechanism of tumor evolution and a driver of resistance to anticancer therapies. The most extensively described clinical settings impacted by such molecular phenomena include neuroendocrine transformation in androgen receptor-dependent prostate adenocarcinoma, and adenocarcinoma-to-neuroendocrine and adenocarcinoma-to-squamous transdifferentiation in epidermal growth factor receptor-driven lung adenocarcinoma, affecting 10%–20% of patients treated with targeted therapy. Recent analyses of human tumor samples and in vivo models of histological transformation have led to insights into the biology of lineage plasticity, including biomarkers predictive of high risk of transformation. However, no clinically available therapies aimed to prevent or revert plasticity are currently available. In the present review, we will provide a biological and therapeutic overview of the current understanding of common and divergent molecular drivers of neuroendocrine and squamous transdifferentiation in tumors from different origins, including descriptive analysis of previously known and recently described molecular events associated with histological transformation, and propose evidence-based alternative models of transdifferentiation. A clear definition of the commonalities and differences of transforming tumors in different organs and to different histological fates will be important to translate molecular findings to the clinical setting.

## Introduction

**Fig. 1:**
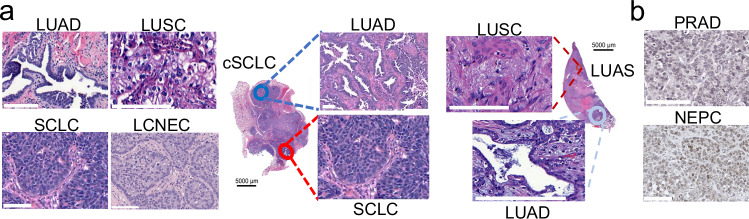
Main histological subtypes involved in histological transformation. **a**, **b** Histological images of the most relevant lung (**a**) and prostate (**b**) cancer subtypes associated with histological transformation. Subtypes include lung adenocarcinoma (LUAD), lung adenosquamous (LUAS), squamous cell lung carcinoma (LUSC), small cell lung cancer (SCLC), large cell neuroendocrine cancer (LCNEC), combined SCLC (cSCLC), prostate adenocarcinoma (PRAD), and neuroendocrine prostate cancer (NEPC). Scale bars, 100 µm.

Lineage plasticity illustrates an ability of cells in one phenotypic state to transition to an alternative developmental pathway. This phenomenon is an increasingly recognized mechanism of tumor evolution, a driver of resistance to anticancer therapies, and remains a clinical conundrum.^1^ The most extensively described clinical setting for such process is histological transformation, defined as the transdifferentiation of tumor cells from one initial histologic subtype to an alternative one (Fig. 1). Lineage plasticity most frequently occurs during treatment with targeted therapy, leading to resistance, and includes neuroendocrine (NE) transformation in androgen receptor (AR)-dependent prostate adenocarcinoma,^2^ estimated to be found in 25% of prostate cancer cases treated with AR-targeted therapies.^3^ Histological transformation is also frequently observed in epidermal growth factor receptor (EGFR)-driven lung adenocarcinoma (LUAD) transforming to NE and squamous carcinomas, with each occurring in 9%–15% of EGFR-driven LUAD treated with targeted therapies.^1,4,5^ However, recent reports suggest that histological transformation may occur as a mechanism of resistance to targeted therapy in many oncogene-driven tumor settings, and even spontaneously in the absence of treatment.^6^

Recent analyses of human tumor samples and in vivo models of histological transformation have led to insights into critical aspects of the biology surrounding lineage plasticity, including biomarkers predictive of high risk of NE transformation.^5,7^ However, to date, there are no clinically available therapies specifically targeted to prevent or suppress lineage plasticity. Critically, because of substantial overlap in the molecular events across tumor types and histological outcomes, approaches to constrain plasticity may be broadly applicable, making “basket trial” like clinical translation both pragmatic and effective.

A clear definition of the commonalities and differences of transforming tumors in different organs and to different histological fates will be important to inform clinical research, define therapeutic targets, and ultimately, optimize care for these patients. In the present review, we will provide an overview of the current understanding of common and divergent molecular drivers of NE and squamous transdifferentiation in tumors from different origins, from biological and therapeutic perspectives. In addition, we perform a joint analysis of previously known and recently described molecular alterations promoting or driving histological transformation to both squamous and NE tumors and propose evidence-based alternative models of transdifferentiation.

## Histological transformations, clinical and molecular context

Besides *EGFR*-mutant LUADs treated with tyrosine kinase inhibitors (TKIs) and metastatic AR-dependent prostate adenocarcinomas treated with enzalutamide, NE transformation has been described in LUADs with alternative genomic contexts and may represent a general mechanism of acquired resistance to other targeted therapies.^1,6,8^ NE-transformed small cell carcinomas are typically rapidly progressive and treatment refractory, leading to prognoses similar to or even worse than de novo small cell lung cancer (SCLC).^7,9,10^ Even if patients with adenocarcinoma at high risk of NE transformation can be identified (i.e., tumors with concurrent *TP53/RB1* inactivation), to date, no therapies are available to effectively constrain plasticity and prevent transformation. The identification of drivers of lineage plasticity and pharmacologically targetable effectors of transformation is unmet clinical needs.

Further increasing the complexity of studying lineage plasticity, the documented time between the initiation of targeted therapy and clinical recurrence is variable. Time on therapy is shorter in patients with combined *EGFR*/*TP53*/*RB1*-mutated LUAD bearing TKI-responsive *EGFR* driver oncogenic alterations and treated with osimertinib.^7^ In prostate cancer, tumor responsive time on androgen deprivation therapy (ADT) in the era of enzalutamide varies as well, but what is consistent is that virtually all NE prostate cancer (NEPC) subtypes proceed first through a castration-resistant prostate cancer (CRPC) intermediate, with CRPC having brief-to-no objective response to ADT.^11^ Moreover, while de novo NEPC remains quite rare (~1% of all diagnosed primaries), it is thought to have a similar, recalcitrant clinical course to transformed NEPC.^12,13^ However, the acquisition of ADT resistance and subsequent development of CRPC do not necessitate transdifferentiation or histological transformation to NEPC.^14^ Critically, it is important to re-emphasize that most targeted therapy recurrences in prostate or lung cancer are not due to NE transformation.^15^

Re-biopsy at clinical recurrence is the most reliable method to assess histological transformation, but the decision to do so varies by institution and patient presentation. Such variability may be partially explained by practicality (i.e., metastatic vs local recurrence) and institutional experience with re-biopsy protocols for any patient with lung cancer (receiving targeted therapy or not). While infrequent, re-biopsy-associated complications remain challenges — specifically, pneumothorax.^16–18^ True matched pairs of pre- and post-histological transformation tumor tissues are still relatively rare. When paired biopsies have been characterized, the protein expression of the adenocarcinoma oncogenic driver in the transformed NE component is typically lost, but some expression is retained in some cases.^19,20^ Of note, similar variations in AR protein production in de novo NEPC and transformed NEPC have been reported.^21^ While most AR-driven prostate cancers result from amplifications and upregulation of protein expression, the genetic diversity of EGFR-driven LUADs includes single and complex point mutations, deletions, fusions, and amplifications.^22–24^ A recent study demonstrated that putative cases of histological transformation were about twice as frequent in patients harboring *EGFR* exon 19 deletions as EGFR L858R alterations.^25^ Such a provocative observation may be most readily supported through parallel datasets in patients of East Asian descent, where LUADs driven by mutant EGFR are more common.^25,26^

As tumor-informed genomic information may help prioritize the likelihood for a tumor to undergo histological transformation, while on targeted therapy, the clinical dilemma of what to do next remains — in patients with tumors likely to transform, should targeted therapy be stopped once transformation is documented, or should it be combined with NE-directed therapy, such as platinum/etoposide doublet chemotherapy? Indeed, recent efforts to introduce SCLC-directed therapy following treatment with osimertinib in *EGFR*-mutant LUAD cases with a high risk of histological transformation have demonstrated that chemotherapy alone does not prevent histological transformation.^27^ In general, detection of NE transformation has historically relied upon available tumor tissue as gold-standard level evidence. Emerging technical advances for the detection of systemic tumor burden utilizing non-invasive approaches, such as circulating tumor DNA (ctDNA),^28^ methylation patterning of ctDNA^28^ and radio-immune conjugate-based detection of NE surface markers,^29,30^ continue to develop and improve sensitivity to detect a spectrum of NE-related cancer subtypes.^31^ This is especially relevant following the US FDA approval of the delta like ligand 3 (DLL3) bispecific T-cell engager (BiTE) tarlatamab for extensive stage SCLC following progression on platinum-based chemotherapy.^32^ It remains to be determined whether DLL3 tumor tissue histology staining score (i.e., H-score), protein production, or RNA expression status is correlated with patient responses to tarlatamab. Interestingly, a recent study collecting circulating tumor cells from patients treated with AR signaling inhibitors demonstrated that detection of multiple NE markers — independent of the loss of AR target gene expression — was sufficient for the detection of NEPC. Such studies encourage future non-invasive testing approaches which may perform best when considering multiple targets/analytes in diagnostic “up assays”.^33,34^

**Fig. 2:**
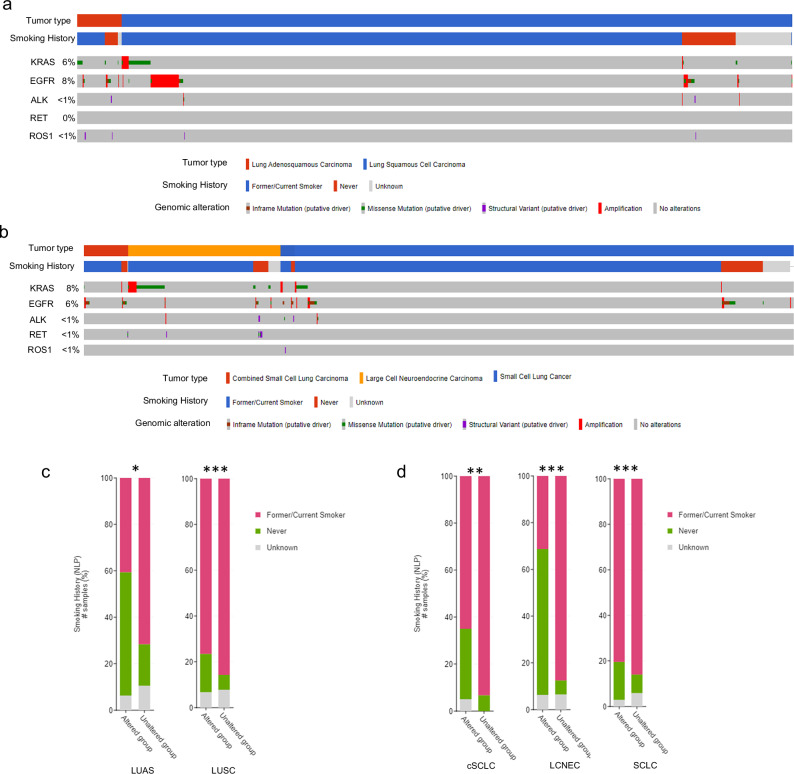
Occurrence of mutations in lung adenocarcinoma (LUAD) drivers in other lung cancer histologies and their association with a low/never-smoking profile. **a**, **b** Oncoprint showing LUAD driver mutations, as profiled by MSK-IMPACT next-generation sequencing, and smoking history of Memorial Sloan Kettering Cancer Center (MSK) lung adenosquamous (LUAS, *n* = 93) and lung squamous carcinoma (LUSC, *n* = 1379) cohorts (**a**), as well as MSK combined histology small cell lung cancer (cSCLC, *n* = 84), large cell neuroendocrine carcinoma (LCNEC, *n* = 223), and small cell lung cancer (SCLC, *n* = 772) cohorts (**b**). cSCLC tumors are defined as tumors containing both SCLC and non-small cell lung cancer (NSCLC Like SCLC tumors) histologic subtypes, where the NSCLC subtype can be LUAD, LUSC, large cell carcinoma (LCC), LCNEC or any other minor NSCLC histologic subtype. **c**, **d** Barplots showing the percentage of different patient smoking profiles for patients with LUAS or LUSC (**c**) or with cSCLC, LCNEC and SCLC (**d**). Altered groups represent patients with tumors harboring mutations in genes that are known LUAD drivers and are associated with a low smoking profile in the LUAD setting (*EGFR*, *ALK*, *RET*, *ROS1*). Unaltered groups represent patients with tumors that do not harbor mutations in the previously mentioned genes. Distribution of smoking history groups was compared using *χ*^2^ test. *P < 0.05, **P < 0.01, ***P < 0.001. Figures were generated in cBioPortal.org^247–249^ with data from the MSK-IMPACT clinical cohort.^250^ Histology annotations were obtained from clinical diagnosis, in the style of real word data (RWD). “*n*” represents the number of patients.

Though most commonly observed in the context of targeted therapy, histological transformation may occur independently of treatment. In lung cancer, ~5% of primary lung tumors present with combined histology, i.e., single tumors showing foci with different histologic subtypes, usually along with areas with intermixed histological phenotypes (Fig. 1).^35,36^ A number of studies confirm shared driver mutations between the histological subtypes found in combined LUAD/SCLC and lung adeno-squamous (LUAS) tumors,^6,37,38^ which supports the occurrence of plasticity rather than the development of two concurrent independent tumors. In this line, known LUAD driver oncogenic alterations can be found (1) in SCLC tumors, including *KRAS* and frequent *EGFR* ( ~ 5%) mutations, as well as *ALK* or *ROS1* translocations; (2) in squamous cell lung cancers (LUSCs),^39,40^ where *EGFR* mutations can be detected in 4%–8% of LUSC patients^41–43^; and also (3) in large cell NE carcinoma (LCNEC) at lower frequencies (Fig. 2a, b). Remarkably, tumors harboring LUAD driver mutations associated with a low/never-smoking profile (*EGFR* mutations and fusions of *ALK*, *RET* or *ROS1*) are enriched in low/never-smoking patients diagnosed with LUAS, LUSC, combined histology SCLC, LCNEC or SCLC^43,44^ (Fig. 2c, d), even if these tumor types are strongly associated with a heavy smoking profile. These results suggest the possibility that such LUSC, LCNEC and SCLC tumors may be a result of lineage plasticity in the absence of treatment.

**Fig. 3:**
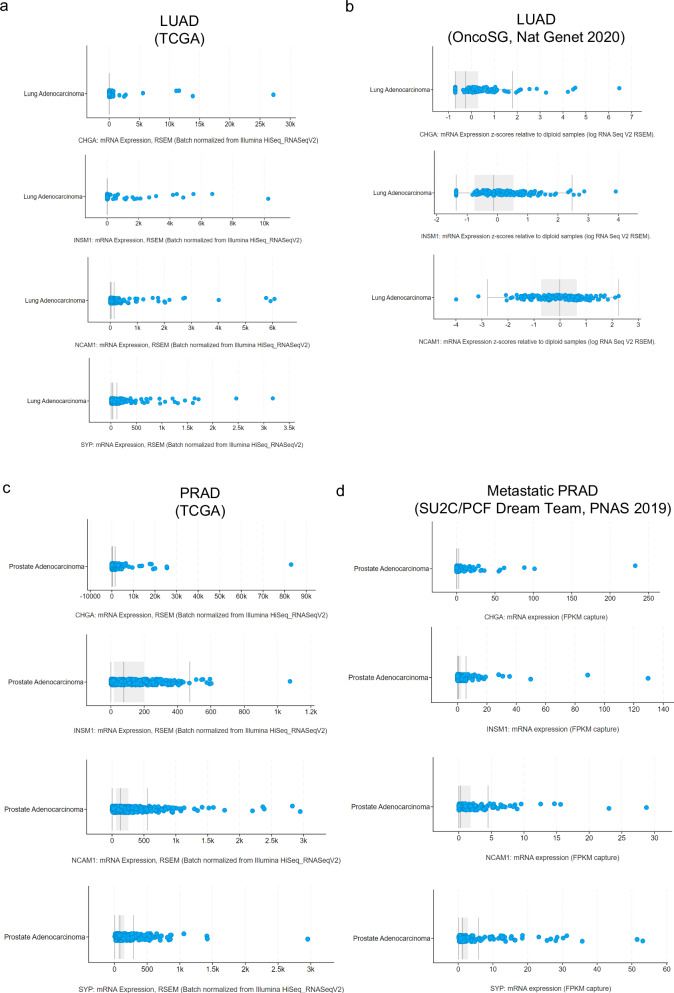
High expression of neuroendocrine (NE) markers in a small subset of lung adenocarcinoma (LUAD) and prostate adenocarcinoma (PRAD) clinical specimens. **a**–**d** mRNA expression levels for key NE markers CHGA, INSM1, NCAM1 and SYP in different LUAD (**a**, **b**) and PRAD (**c**, **d**) cohorts, including The Cancer Genome Atlas^251^ (TCGA, *n* = 566) (**a**, **c**), OncoSG^26^ (*n* = 305) (**b**) and SU2C/PCF Dream Team^47^ (*n* = 444) (**d**). Each blue dot represents a sample. Plots represent expression values per sample, as pre-processed and normalized in the particular study of interest. Expression values are shown to illustrate interpatient heterogeneity and a subset of tumors exhibiting differentially higher expression. Figures were generated in cBioPortal.org.^247–249^

Like SCLC tumors, LCNECs also exhibit a particularly high frequency of *KRAS* mutations, another driver genomic alteration in LUAD, occurring in > 20% of LCNECs. LCNECs with such LUAD driver mutations have been classified as non-small cell lung cancer (NSCLC)-like LCNEC,^45^ and it cannot be ruled out that some of these may be histological transdifferentiation cases. Indeed, a minor proportion of LUADs without NE morphology present a certain degree of NE features at diagnosis and may be enriched for *EGFR* mutations and *ALK* translocations,^46^ and similarly, a small percentage of treatment-naive advanced prostate adenocarcinomas exhibit NE differentiation^47^ (Fig. 3). These observations indicate that targeted therapy may not be essential for histological transformation, and thus, that the convergence of the appropriate molecular alterations in the tumor may lead to spontaneous transformation under no treatment selection pressure.

Poorly differentiated tumors might similarly be a result of plasticity. In these tumors, the features that allow histological classification may be relatively focal, thus requiring examination of several tissue blocks to establish a particular pattern of differentiation.^48^ Undifferentiated tumors exhibit a stem-like state^49^ and may represent intermediate dedifferentiated states with the potential to transition to a different histological subtype or may be anchored in a deprogrammed state. Indeed, poorly differentiated solid tumors exhibit expression of a number of molecular factors previously involved in histological transdifferentiation,^6,38^ including stemness-related transcription factors like SRY-box transcription factor 2 (SOX2) and MYC or activation of the PRC2 epigenetic remodeling complex.^49–51^

## Emerging pathways regulating tumor cell plasticity

A tumor’s potential for lineage plasticity is influenced not only by its genomic alterations but also by the underlying developmental and epigenetic landscape of the initiating cell. This foundational state, shaped by the cell of origin, can prime tumor cells for specific responses to oncogenic stress or therapy. However, plasticity is not dictated by developmental identity alone. A growing body of evidence highlights a series of molecular pathways that, in the right molecular context, contribute to phenotypic switching under selective pressure, often overriding or reprogramming lineage constraints. In this section, key signaling and regulatory mechanisms are outlined, including *TP53*/*RB1* co-inactivation, *MYC* amplification, AKT/mTOR pathway, FGFR signaling, Wnt signaling, epigenetic regulators, immune modulation, inactivation of *STK11*/*KEAP1,* and the APOBEC-mediated mutagenesis.

### Co-inactivation of TP53 and RB1

TP53 and RB1 are central regulators of cellular differentiation and plasticity, especially in cancer biology, where their loss fosters tumor progression and therapeutic resistance. TP53, known as the “guardian of the genome”, promotes lineage fidelity by suppressing dedifferentiation and enhancing terminal differentiation. Loss of TP53 function disrupts these processes, increasing cellular plasticity and enabling the acquisition of undifferentiated, stem-like states that drive tumor initiation and progression. RB1, a central cell cycle regulator, enforces lineage-specific differentiation programs through interactions with transcription factors and chromatin remodeling proteins. Disruption of RB1 function permits re-entry into the cell cycle, enhancing plasticity and contributing to tumor heterogeneity. In advanced cancers, such as metastatic CRPC, *RB1* loss is associated with phenotypic plasticity and aggressive clinical outcomes. Genomic analyses reveal that *RB1* and *TP53* deletions are enriched in metastatic CRPC, particularly in low prostate-specific antigen populations, correlating with increased visceral metastasis and shorter survival.^52,53^ The combined loss of *TP53* and *RB1* is mechanistically linked to lineage plasticity and antiandrogen resistance. In *TP53-* and *RB1-*deficient prostate cancers, tumors evade AR-targeted therapies by transitioning from AR-dependent luminal cells to AR-independent basal-like phenotypes. This phenotypic shift is driven by upregulated SOX2, a reprogramming transcription factor, and can be reversed by restoring TP53 and RB1 function or inhibiting SOX2 expression.^51,52,54^ Together, TP53 and RB1 maintain differentiation and suppress plasticity; their disruption creates a microenvironment conducive to tumor evolution, with implications for disease aggressiveness and resistance to therapy.^55^

While *TP53* and *RB1* loss establishes a permissive state for transformation,^56,57^ it is insufficient to induce complete transformation into a malignant state. Additional oncogenic alterations, such as KRAS or MYC activation, *PTEN* loss, or changes in the tumor microenvironment (TME), are required to complete the transition to malignant phenotypes.^58–62^ In LUAD, concurrent mutations in *TP53* and *RB1* facilitate histologic transformation to SCLC, but additional mutations and amplifications in *PIK3CA*, *MYC*, and *NOTCH* are also critical for the complete transformation process.^58^ Similarly, in prostate cancer, loss of *TP53* and *RB1* leads to lineage plasticity and JAK/STAT activation, giving rise to therapy-resistant, stem-like subclones with suppressed AR and luminal markers. Single-cell RNA sequencing has revealed distinct subpopulations of this kind, exhibiting suppressed expression of luminal lineage markers and increased expression of stem-like and epithelial-to-mesenchymal transition (EMT) genes.^63–65^ Notably, the process of transformation is not typically a clonal sweep but involves multiple subclones with distinct genetic alterations. This intratumoral heterogeneity allows for the selection of subclones that can adapt to therapeutic pressures and contribute to NE transformation.^6^

Several recent studies on JAK/STAT inflammatory signaling highlight the role of *TP53* and *RB1* loss in lineage plasticity, showing that organoid models with these deletions enter a smoldering EMT-like state but fail to achieve full NE transition.^63,65–67^ This incomplete transformation suggests that additional microenvironment signals, such as JAK/STAT and FGFR activation, are required. In vivo, genetically-engineered mouse models (GEMMs) reveal further insights; while PtR (*PTEN*/*RB1* loss) models exhibit limited plasticity, PtRP (*PTEN*/*RB1*/*TP53* loss) models show marked NE phenotype, demonstrating how *TP53* loss synergizes with *RB1*/*PTEN* deletions, emphasizing the interaction between genetic alterations and systemic factors. Another recent study^68^ highlights the critical role of in vivo conditions in driving full NEPC transformation using the PARCB (*TP53* and *RB1* abrogation, *AKT* overactivation, and *MYC* and *BCL2* overexpression) genetic model. The study shows that primary basal prostate epithelial cells, modified with key oncogenic drivers, underwent partial reprogramming when cultured in an organoid system. Transformation to fully differentiated NEPC required in vivo engraftment to immunodeficient mice, where transplanted cells formed tumors with squamous cell prostate cancer histological features, including high nuclear-to-cytoplasmic ratios, frequent mitoses, and uniform NE differentiation marker expression. This underscores the necessity of non-cell-autonomous signals such as TME signals in driving full transformation. Interestingly, despite successful NEPC transformation in immunodeficient NSG-engrafted xenografts, limited metastasis was observed, suggesting the absence of essential immune or stromal cues critical for tumor dissemination. Collectively, these studies indicate that *TP53*/*RB1* loss is a critical but insufficient driver of NE transformation. Full lineage conversion requires cooperative genetic alterations and signals from the TME to reprogram prostate adenocarcinoma into NEPC.

### The role of MYC in plasticity

The amplification of *MYC* in relapsed or recurrent SCLC is driven by its critical role in tumor plasticity, heterogeneity, and resistance. Studies reveal that *MYC* is frequently amplified on extrachromosomal DNA (ecDNA), a structure enabling exceptionally high transcriptional activity and rapid genomic heterogeneity through random mitotic segregation. This amplification is often acquired under therapeutic pressure, as seen in patient-derived xenografts (PDXs) of treatment-resistant disease, where MYC drives cross-resistance to DNA-damaging therapies.^69,70^ The challenge, therefore, may lie in targeting these adaptive mechanisms associated with plasticity in MYC-overexpressing, treatment-resistant cells. The dual involvement of MYC in NE transformation and squamous transdifferentiation^38^ adds further complexity. In SCLC, MYC can drive NE phenotypes, but under certain contexts, it might also promote squamous characteristics, potentially influencing tumor heterogeneity. In light of these observations, assessment of MYC expression by immunohistochemistry might help identify patients at risk for transformation and relapse.^38^

### AKT/mTOR pathway

Evidence suggests that, in the right molecular context, activation of the PI3K/AKT pathway may act as a driver of lineage plasticity. In the lung setting, AKT/mTOR activation has been associated to transdifferentiation of LUAD to acquire LUSC features. This process is not solely driven by genomic alterations but also involves transcriptional reprogramming and epigenetic changes. Combined overactivation of AKT and MYC in LUAD models synergistically induces squamous markers such as P40, with further augmentation under the selective pressure of EGFR inhibition.^38^ Similarly, in prostate cancer, the AKT/mTOR pathway has been associated to lineage plasticity, facilitating the transition from AR-dependent adenocarcinoma to AR-independent states like NEPC.^68^ This transition often follows ADT and is closely linked to resistance mechanisms. The activation of this pathway supports cell survival, interacts with AR signaling, and modulates epigenetic regulators like EZH2 to enable phenotypic shifts.^71,72^ In both cancer types, the AKT/mTOR pathway functions as a pro-survival signaling mechanism that enhances cell proliferation, supports cellular transitions, and underpins therapy resistance and disease progression.

### Epigenetic factors

Epigenetic reprogramming underpins lineage plasticity and therapy resistance in prostate and lung cancers by facilitating transitions to alternative, treatment-resistant phenotypes. This plasticity is driven by dynamic changes in chromatin structure and transcriptional networks, enabling tumor cells to evade therapeutic pressures and adopt new lineages. Key drivers include EZH2, a histone methyltransferase that silences lineage-specific genes through H3K27me3 marks. EZH2 plays a pivotal role in supporting NE differentiation and EMT, both of which contribute to tumor progression and resistance mechanisms.^54,73–76^ A recent clinical study showed that combining mevrometostat, an EZH2 inhibitor, with enzalutamide improved progression-free survival and response rates in metastatic CRPC compared to enzalutamide alone.^77^ FOXA1, a pioneer transcription factor, facilitates chromatin remodeling and regulates AR-dependent transcription. In prostate cancer, *FOXA1* is frequently mutated. Studies annotating the landscape of *FOXA1* mutations from 3086 human prostate cancers, defined two hotspots in the forkhead domain Wing2 and the highly conserved DNA-contact residue R219. Wing2 mutations are detected in adenocarcinomas at all stages, whereas R219 mutations are enriched in metastatic tumors with NE histology. These mutations in *FOXA1* alter its chromatin binding activity, shifting transcriptional programs toward NE states, particularly under ADT.^78,79^ Beyond genetic alterations, FOXA1 can undergo cistromic reprogramming in NEPC, even in the absence of mutation. In this context, FOXA1 cooperates with transcription factors such as achaete-scute family bHLH transcription factor 1 (ASCL1) and NKX2-1 to activate NE-specific enhancers, supporting an NE phenotype.^79^ In parallel, FOXA2 promotes NE differentiation by regulating chromatin accessibility and activating pathways such as the KIT pathway.^79^ FOXA2 facilitates transcriptional reprogramming by cooperating with JUN/AP-1 complexes at lineage-specific enhancers, enabling chromatin remodeling and plasticity. The interaction between FOXA2 and the epigenetic enzyme LSD1 further supports its chromatin binding activity, and inhibition of LSD1 disrupts FOXA2-driven transcriptional programs, thereby reducing lineage plasticity.^80^

In prostate cancer, the loss of CHD1, a chromatin remodeler, disrupts chromatin integrity, leading to increased transcriptional plasticity and resistance to AR-targeted therapies.^81^ DNA methylation is another critical epigenetic mechanism driving lineage plasticity. This process is regulated by DNA methyltransferases (DNMT1, DNMT3A, DNMT3B) and TET proteins (TET1, TET2, TET3), which modify the methylation landscape to enable tumor adaptability. A recent study demonstrated that *ZNF397* loss triggers a TET2-driven transformation of the 5-hydroxymethylcytosine landscape into a plastic state characterized by AR independence and multilineage potential, further contributing to therapy resistance.^82^ In lung cancer, alterations in the SWI/SNF complex, including mutations in *SMARCA4*, destabilize chromatin and facilitate lineage switching, including adenocarcinoma-to-squamous or NE transformation.^83^ These epigenetic regulators mediate the chromatin remodeling and transcriptional reprogramming necessary for transitions such as adenocarcinoma-to-squamous differentiation and NE transformation. Under therapeutic pressures, tumor cells exploit these transitions to adopt alternative lineages that evade treatment. Together, these findings underscore the central role of epigenetic regulators in shaping lineage plasticity and therapy resistance in prostate and lung cancers. Targeting these mechanisms could provide novel therapeutic opportunities to prevent lineage transitions and improve treatment outcomes.

### FGFR signaling

FGFR signaling plays a pivotal role in regulating lineage plasticity and tumor development by influencing cellular trafficking and oncogenic activation. The LIS1/NDE1 complex is crucial for FGFR intracellular trafficking, stability, and recycling, ensuring proper signal strength and localization.^84^ Dysregulation of FGFR signaling can alter lineage-specific programs and drive phenotypic shifts, with significant implications for cancer lineage plasticity. For instance, in SCLC, FGFR1 oncogenic activation reveals an alternative cell of origin and influences tumor phenotype, promoting low-grade NE lesions in specific contexts while impairing the development of typical SCLC.^85^ These findings highlight the nuanced roles of FGFR signaling in determining tumor lineage and phenotype. In prostate cancer, Chan et al.^65^ underscores the importance of FGFR and JAK/STAT signaling as early and essential mediators of plasticity. These pathways drive basal-luminal mixing, EMT, and eventual NE transformation. In Trp53/Rb1-deficient organoids and GEMMs, both pathways are activated and further enhanced under therapeutic pressures, such as androgen deprivation or AR inhibition. Functional experiments have revealed that FGFR activation promotes plasticity-associated phenotypes, including changes in cellular identity and morphology, while JAK/STAT signaling amplifies transcriptional programs critical for the transition to treatment-resistant states. Collectively, these findings position FGFR signaling as a key regulator of cancer lineage plasticity and a potential therapeutic target. Understanding the interplay of FGFR signaling with pathways like JAK/STAT may provide new insights into the mechanisms driving tumor progression and resistance, particularly in cancers characterized by high plasticity and phenotypic heterogeneity.

### Immune dysregulation

Lineage plasticity during NE and LUSC transformation actively suppresses the immune response, allowing tumors to evade immune surveillance and thrive in hostile microenvironments. These transformations are characterized by profound transcriptional and epigenetic reprogramming as well as extrinsic microenvironmental changes. A key feature of this transformation is the downregulation of major histocompatibility complex class I (MHC-1) molecules, which impairs antigen presentation and reduces visibility to cytotoxic T lymphocytes. This phenomenon has been observed in NEPC and squamous-transformed leukemia. In these models, promoter hypermethylation was responsible for MHC-1 loss, creating an immunologically “cold” tumor state.^6,38^ Recent studies also highlight hypoxia as a critical driver of immune dysregulation and lineage plasticity in prostate cancer. Hypoxia stabilizes HIF1a, which cooperates with transcriptional regulators such as ONECUT2 and SMAD3 to promote NE differentiation and downregulation of MHC-1 molecules. By linking transcriptional reprogramming with immune escape, hypoxia-driven signaling creates a permissive environment for NE transformation and therapy resistance.^6,38,86^ As described above, the ectopic activation of JAK/STAT signaling in tumor epithelial cells,^63,65–67^ a pathway commonly associated with immune and inflammatory responses, plays a pivotal role in these processes. The chronic activation of JAK/STAT not only disrupts normal immune surveillance but also drives the production of inflammatory mediators that reshape the TME. This inflammatory signaling promotes an immunosuppressive milieu that inhibits effective immune cell infiltration and activation. Furthermore, the interplay between JAK/STAT signaling and epigenetic alterations reinforces these immunosuppressive changes, fostering a microenvironment that promotes therapy resistance and tumor progression. These multifaceted changes result in a highly immunosuppressive TME that facilitates tumor growth, enables evasion of immune-mediated destruction, and may contribute to plasticity and resistance to therapies targeting the AR and other key pathways in prostate cancer.

### STK11/KEAP1 inactivation

Genomic alterations of the serine/threonine kinase *STK11* (also known as *LKB1*) and of the adaptor subunit of Cullin-based E3 ubiquitin ligase *KEAP1*, which are often co-occurring, have been detected in different tumor types and associated with tumorigenesis and poor prognosis.^87–90^ KEAP1 acts as a redox sensor that gets inactivated under stress, leading to the upregulation of NRF2, a transcription factor triggering transcriptomic reprogramming in response to stress.^91^ Interestingly, *KEAP1* inactivation has been extensively involved in stemness in different settings, including hematopoiesis^92^ and lung, head and neck, and breast tumors, among others.^40,92,93^

STK11 is physiologically implicated in energy homeostasis as an upstream kinase of the AMP-activated protein kinase (AMPK) pathway. This tumor suppressor is frequently mutated in human cancers such as lung, cervical, ovarian, skin, pancreas, kidney, and gastrointestinal tumors.^94,95^ *STK11* inactivation has been associated with loss of differentiation and increased stem-like features in lung, gallbladder, and glioblastoma tumors.^96–98^ In the lung cancer setting, where the gene has been most extensively studied, the dedifferentiation induced by *STK11* inactivation is mediated by upregulation of stemness factors such as Nanog, inhibition or downregulation of Notch signaling,^98^ and downregulation of salt-inducible kinase (SIK) family members and the AT2 lineage-defining factor C/EBPα,^99^ as reported in different Kras-driven LUAD GEMMs. Indeed, LKB1 has been shown to play a role in histological heterogeneity in a Kras-driven LUAD GEMM, where *Lkb1* inactivation led to the formation of not only LUADs, but also LUSC and large cell carcinomas.^100^ *Lkb1* inactivation induced chromatin alterations, including the loss of H3K27me3 and gain of H3K27ac and H3K4me3 particularly at squamous lineage genes, including *Sox2*, *ΔNp63*, and *Ngfr*.^101^ Importantly, even if *LKB1* mutations are common in human LUSC ( ~19%), they also occur frequently in LUADs ( ~34%),^100^ suggesting that *LKB1* inactivation may be an enabler of plasticity and histological transformation, rather than a driver in this process. In line with these results, different cells of origin may display different efficiencies of squamous differentiation, where bronchioalveolar stem cells and club cells may be the most prone to transformation to a squamous phenotype.^102^ Of note, the adeno-to-squamous transition has been thoroughly characterized in *Kras*/*Lkb1*-mutant GEMMs, being advanced most significantly by Hongbin Ji’s group.^100,103–105^

**Fig. 4:**
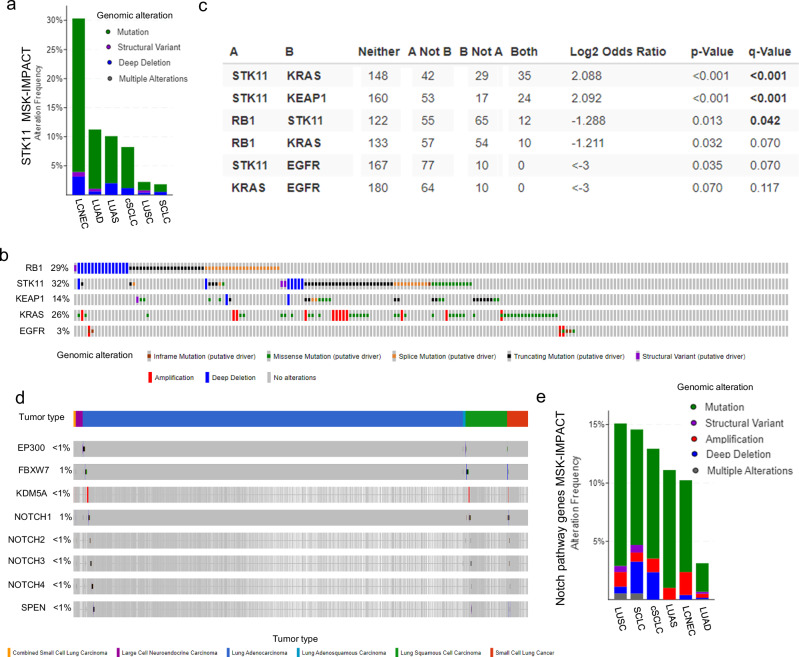
Genomic alterations in *STK11* and genes within the Notch signaling family in different lung cancer histologies. **a** Frequency of driver mutations in *STK11* in the different lung cancer histologic subtypes, large cell neuroendocrine carcinoma (LCNEC), lung adenocarcinoma (LUAD), lung adenosquamous (LUAS), combined small cell lung cancer (cSCLC), squamous cell lung carcinoma (LUSC) and SCLC. **b** Oncoprint showing mutations in genes of interest in the LCNEC Memorial Sloan Kettering Cancer Center (MSK) cohort. **c** Co-occurrence or mutual exclusion of mutations in the LCNEC MSK cohort, quantifying how strongly the presence or absence of alterations in A are associated with the presence or absence of alterations in B in the selected samples. **d** Oncoprint showing mutations in genes related to the Notch signaling pathway in the lung cancer MSK cohort, divided by histological subtypes. **e** Frequency of genomic alterations in genes related to the Notch pathway (see **d**) in the different lung cancer histological subtypes from the MSK cohort. Odds Ratio = (Neither × Both)/(A Not B × B Not A). *p*-values are derived from two-sided Fisher Exact Test and *q*-values were derived from a Benjamini-Hochberg false discovery rate correction procedure. Log_2_ratio > 0: tendency towards co-occurrence; log_2_ratio ≤ 0: tendency towards mutual exclusivity. *q*-value < 0.05: significant association. Figures were generated in cBioPortal.org^247–249^ with data from the MSK-IMPACT clinical cohort.^250^ Histology annotations were obtained from clinical diagnosis, in the style of RWD. MSK LUAD (*n* = 9789), LUAS (*n* = 93), LUSC (*n* = 1379), cSCLC (*n* = 84), LCNEC (*n* = 223), and SCLC (*n* = 772) cohorts are depicted. “*n*” represents the number of patients.

Remarkably, inactivating mutations of both *STK11* and *KEAP1* are common in LCNEC, where they frequently co-occur alone or in combination with *KRAS* mutations (Fig. 4a, b). LCNECs are considered intermediate entities between NSCLC and SCLC, harboring frequent LUAD-associated genomic alterations, but exhibiting NE features.^45,106^ Interestingly, recent molecular characterizations of these rare tumors converge into a classification driven by genomic events, which distinguishes SCLC-like LCNEC, enriched for *RB1* mutations, and NSCLC-like LCNEC, enriched for *STK11* and *KEAP1* mutations. The fact that mutations in *RB1* and *STK11* are mutually exclusive in these tumors (Fig. 4c), and the role of both in maintaining LUAD differentiation, further supports that both tumor suppressors might be master regulators of plasticity, and that LCNECs, or a subset of them, may potentially be cases of histological transformation.

### Wnt pathway

The Wnt/β-catenin pathway plays critical roles in embryonic development and adult tissue homeostasis^107^ and has been reported to have pro-oncogenic roles in different tumor types, including gastrointestinal carcinomas, leukemia, melanoma, and breast cancers.^108^ One of the most studied roles of this signaling pathway includes the maintenance of stemness through a variety of mechanisms, including TERT overexpression^109^ and induction of dedifferentiation.^110^ Multi-omic analyses of lung tumor clinical specimens undergoing histological transformation revealed upregulation of genes involved in the Wnt signaling pathway, as well as upregulation of β-catenin at the protein level, occurring convergently in both LUSC and NE transformations.^6,38^ A recent study integrating ATAC-sequencing and RNA-sequencing data from patient-derived organoids (PDOs), PDXs, and cell lines identified four subtypes of CRPC and predicted key transcription factors of each. Among these, the so-called “CRPC-WNT” subtype was characterized as AR-negative/low, Wnt-dependent, and driven by TCF/LEF transcription factors. Even if the “CRPC-WNT” and the NE-like “CRPC-NE” subtypes represented distinct molecular subsets,^111^ components of the Wnt signaling pathway have been implicated in promoting NE-like features and lineage plasticity in the prostate. AR inhibition leads to Wnt signaling upregulation through the downregulation of Wnt ligand secretion mediator (WLS), a transcriptional regulator and major driver of the NE phenotype.^112^ In line with this, other effectors of the Wnt pathway, such as TCF4, FOXB2-Wnt7B and Wnt11, have been reported to facilitate NE transformation in the prostate, by inducing a transcriptional program favoring neuronal differentiation.^113–115^

### Notch pathway

The notch pathway controls cell fate decisions critical for proper organ development during embryogenesis and homeostasis in adult organisms. This pathway relies on cell surface receptors and ligands for activation that leads to receptor cleavage. The cleaved receptor then acts as a transcriptional regulator and controls differentiation.^116^ Notch signaling may have both oncogenic and tumor suppressor effects depending on the molecular context and has been reported to be incompatible with the NE phenotype. Specifically, Notch signaling is a strong suppressor of NE differentiation via upregulation of the transcription factors HES1 and REST, which suppress expression of the master NE regulator ASCL1 and other NE genes in prostate cancer and pancreatic carcinoma.^116^ In line with these results, Notch signaling downregulation was observed at early stages of NE transformation in the lung, specifically in pre-transformation LUAD, as compared to control, never-transformed LUAD clinical specimens.^6^ Similarly, in the prostate setting, it has been reported that hypoxia-driven Notch signaling inactivation is able to induce NE differentiation.^117^ Consistently, genomic alterations in Notch family genes are common in NE tumors (Fig. 4d, e).

Remarkably, even if Notch signaling is incompatible with the NE phenotype, non-NE SCLC subtypes exhibit activation of Notch signaling,^118^ whose induction has been described in the transition of NE to non-NE SCLC observed during disease progression or development of chemotherapy resistance.^119,120^ These observations suggest that Notch signaling may have a role in SCLC biology after NE transformation. Indeed, different works have described intratumoral heterogeneity within SCLC tumors, containing both NE and non-NE populations. In these, expression of Notch ligands in the NE compartment activates Notch receptors in the non-NE compartment, and the interaction of both populations may represent a selective advantage in terms of disease progression and metastasis.^121,122^ However, a recent study suggests that conditional expression of a single inhibitory Notch ligand during SCLC development does not necessarily anchor SCLC to a pure NE state, where the authors observed similar heterogeneity of NE and non-NE tumor cell types.^123^ Such heterogeneity is reminiscent of the occurrence of persistent, even if minor, adenocarcinoma components in some transformed NE tumors,^6^ where similarly NE and non-NE cells may potentially be cooperating with pro-oncogenic consequences.

Although Notch signaling appears to be at odds with NE tumors, RNA sequencing analyses on clinical specimens undergoing adenocarcinoma-to-squamous transformation indicate a transcriptomic profile compatible with Notch signaling activation.^38^ Certainly, Notch signaling has been associated with poor prognosis and stemness in NSCLC.^124^ Nonetheless, Notch signaling activation may have differential effects in different molecular or histological contexts, with oncogenic effects in LUAD but potential tumor suppressive effects in LUSC as well as in other squamous tumors.^124^ Interestingly, we observed increased frequency of mutations in members of the Notch signaling pathways in LUAS tumors relative to LUAD, and further enrichment of mutations in these genes in LUSC tumors (Fig. 4d, e). Remarkably, mutations in genes related to Notch signaling are very common ( ~70%) in other squamous tumors, such as head and neck cancer,^125^ further associating the dysregulation of this pathway with squamous differentiation. The role of Notch signaling in histological transformation remains undefined and in need of additional study.

### APOBEC-mediated hypermutation

In addition to single genetic alterations, large-scale genomic changes and mutational processes, such as APOBEC-mediated hypermutation and whole-genome doubling, are critical drivers of lineage plasticity and therapy resistance. APOBEC enzymes, a family of cytidine deaminases, introduce mutations that can lead to widespread genomic instability, contributing to tumor heterogeneity and adaptive evolution. Enrichment of APOBEC hypermutation signatures has been observed in cohorts of patients with SCLC tumors undergoing phenotypic transformation, highlighting its role in facilitating tumor plasticity under therapeutic pressure.^19^ Recent findings have elucidated the mechanisms underlying APOBEC-driven mutagenesis, with the loss of SYNCRIP, an RNA-binding protein that inhibits APOBEC3B activity, identified as a key contributor.^126^ The loss of SYNCRIP unleashes unchecked APOBEC3B activity, leading to extensive mutational processes that target critical regulatory genes. Among these are *FOXA1*, *EP300*, and *STAT3 *— genes central to the maintenance of transcriptional networks. Mutations in these genes disrupt normal regulatory functions, enabling transcriptional reprogramming that fosters lineage transitions and confers resistance to therapy.^126^ Moreover, APOBEC-mediated mutagenesis is thought to generate genetic diversity that equips tumors to adapt to changing selective pressures, including those imposed by therapeutic interventions. This mutational process can amplify genomic alterations that promote epigenetic reprogramming and phenotypic plasticity, further reinforcing a feedback loop that drives therapy resistance and tumor progression. Understanding the interplay between APOBEC activity and lineage plasticity may reveal novel therapeutic vulnerabilities to overcome resistance mechanisms.

## Influence of cell of origin on lineage plasticity

Evidence suggests that the cell of origin plays a significant role in lineage plasticity during cancer transformation by introducing a bias toward specific histological fates. However, this influence is not absolute, as molecular and environmental factors often dominate the determination of the final histological state, allowing flexibility in lineage transitions.^127^ This interplay between intrinsic lineage programming and extrinsic molecular cues underscores the complexity of tumor plasticity. In prostate cancer, tumors predominantly display a luminal phenotype. Previously, basal cells were identified as the cell of origin for prostate cancer,^128,129^ but increasing evidence implicates luminal cells, particularly luminal progenitor cells (LPs), as a preferred cell of origin. LPs with stem-like properties appear critical for both tumor initiation and progression.^130^ A lineage tracing study has identified a rare population of castration-resistant Nkx3-1-expressing luminal cells that function as bipotent stem cells during regeneration and serve as efficient targets for oncogenic transformation, rapidly forming carcinoma following *Pten* deletion.^131^ Functional studies using human prostate epithelial cells transplanted into immunodeficient mice, as well as organoid-based transformation assays further support this paradigm. In these models, basal and luminal cells isolated from primary benign human prostate tissue were transduced with defined oncogenes (e.g., *MYC*, *AKT*, *ERG*), and then either xenografted into NSG mice or cultured in 3D organoid systems. These studies revealed that while basal cells can also initiate prostate cancer when transformed, they often give rise to tumors with more poorly differentiated phenotypes, whereas luminal-derived tumors retain glandular morphology and strong AR activity.^128,129^ This indicates that the epithelial cell of origin shapes not only tumorigenic potential but also differentiation state. Additionally, the transcriptional and epigenetic landscape of LPs renders them particularly susceptible to lineage plasticity and NE reprogramming under therapeutic pressures such as ADT. Studies investigating the cell of origin for prostate cancer using tissue regeneration models and GEMMs have yielded different results, highlighting the influence of model context. These differences indicate the critical role of microenvironmental components in shaping the susceptibility of specific epithelial lineages to transformation.^132^ These findings underscore that both intrinsic lineage identity and the microenvironmental context of transformation critically influence plasticity trajectories and therapeutic resistance in prostate cancer.

In lung cancer, similar principles apply, with different progenitor cells showing varying propensities for transformation into specific histological subtypes.^119,133^ For example, AT2 cells frequently give rise to adenocarcinomas, while basal cells are more likely to transform into squamous cell carcinoma. Additionally, club cells have been implicated in contributing to SCLC. These cell-specific biases reflect intrinsic lineage determinants that shape tumor histology in response to oncogenic or therapeutic pressures. Despite these similarities, notable differences exist between the behavior of NE cells in the lung and prostate. In the lung, NE cells differentiate at specific anatomical sites, such as branch points, and exhibit a unique migratory behavior known as “slithering”, which enables them to form neuroepithelial bodies.^134^ In contrast, NEPC cells emerge more broadly under stress conditions, such as ADT, and arise from luminal progenitors without requiring notable migration. This difference in NE cell behavior reflects tissue-specific adaptations in response to environmental and molecular cues. These observations suggest that while the cell of origin establishes a foundation for lineage plasticity, the eventual histological outcome is shaped by a complex interplay between intrinsic lineage programming and external molecular influences. Understanding these mechanisms is crucial for identifying vulnerabilities that can be targeted to limit lineage plasticity and overcome therapy resistance.

## Preclinical models to interrogate histological transformation and lineage plasticity

Several important questions surrounding mechanisms and timelines in histologic transformation remain unanswered but can be directly addressed using currently available models. First, it is unclear whether inhibition of oncogenic drivers in tissue types other than the lung or prostate will display similar frequencies of histologic dedifferentiation as mechanisms of acquired resistance to said targeted therapies. For example, it is clear that loss of *Rb1* and *Trp53* alone is insufficient to permit the transition or adoption of an NE fate in an LUAD tumor when the oncogenic driver, such as *Kras(G12D)*, is still active. While not the primary intention of the study, recent work utilizing an *Rb1* allele that could be inactivated and then restored had shown that triple mutant *Kras*, *Trp53*, *Rb1* (KPR) mice develop aggressive, metastatic LUAD with features of dedifferentiation, which is often not observed in the KP LUAD model until much later in tumor development.^135^ However, the histologic characterization of these lesions was neither NE nor consistent with SCLC. Whether Kras(G12D) or pan-Ras inhibition encourages NE transformation remains open questions. Recent studies on mechanisms of Kras inhibitor resistance in LUAD have suggested that lineage plasticity towards a more squamous fate (if *Lkb1* is deactivated) or perhaps even dedifferentiation towards an alternative alveolar state (i.e., AT1-like) are more common mechanisms of acquired resistance than NE differentiation.^104,136,137^ In line with the models mentioned above, a role for *Rb1* genetic restoration in the maintenance of an NE state would be directly addressable. Similar tools have been instrumental in deciphering a role for *Trp53* and *Lkb1* inactivation during multiple stages of tumorigenesis and in the maintenance of a transformed state.^99,138^ Further, whether expanded therapeutic application of mutant-selective and/or pan-Ras inhibitors in pancreatic, colorectal/gastrointestinal, and lung cancers result in NE transformation remains to be seen.^139–141^

To the best of our knowledge, most studies describing model systems to study histologic transformation have failed to observe fully differentiated NE transformation outside of a host animal or patient, and the reason for this remains an open question. For example, isolation of reporter-positive prostate cancer cells from the PtRP mouse model of luminally-derived prostate cancer display progressive downregulation of the AR and eventual dedifferentiation to NEPC but fail to fully commit to an Ascl1^+^ NE state ex vivo.^65^ This appears to be similarly true in other systems utilizing engineered human cells, requiring in vivo engraftment into well-perfused sites (i.e., renal capsule) or sub-cutaneous engraftment.^68^ Indeed, in our own experience, working with organoid-derived cultures from a GEMM that combines the conditional expression of *Myc*, *rtTA3*, and *tdTomato* with the loss of *Rb1* and *Trp53*, along with a doxycycline (Dox)-inducible, oncogenic *EGFR* transgene (ERPMT model), we observed that LUAD histological transformation to SCLC failed to adopt an NE fate following de-induction of the EGFR oncogenic driver.^142^

GEMMs offer a critical platform for investigating microenvironmental influences on lineage plasticity. Complementary preclinical models, such as PDOs, PDXs, and co-culture systems, enable investigation of TME interactions in human contexts. Notably, a panel of 22 prostate cancer PDOs generated at MSK, along with four NEPC PDOs (WCM154, WCM155, WCM1262, WCM1078) and their corresponding xenografts, constitute a functionally annotated, lineage-diverse model system,^143,144^ that serve as an effective platform for dissecting the transcriptional and epigenetic mechanisms underlying lineage plasticity. Co-culture systems using these organoids with immune components can be a useful model system for the study of tumor–immune interactions and the microenvironment. A longitudinal study using the *Pten*^*(i)pe–/–*^ mice, which carry a prostate-specific deletion of *Pten*, and *Pten/Hif1a*^*(i)pe−/−*^ mice, with combined *Pten* and *Hif1a* deletion, demonstrated that HIF1a is essential for initiating the transcriptional programs associated with lineage plasticity, immune evasion, and NE differentiation in the context of *Pten* loss.^86^

Meanwhile, PPR (*Pten*, *TP53*, and *RB1* loss) and PRP (*Pten*, *RB1*, and *TP53* loss) models have been used to underscore the pivotal role of combined genetic alterations in driving lineage plasticity. Loss of *RB1* and *TP53*, along with *Pten* deletion, facilitates the transition of prostate cancer cells from AR-dependent luminal states to basal-like or NE phenotypes. One of the critical mediators of this plasticity is Sox2, a transcription factor known for its role in embryonic stem cell maintenance. Sox2 reactivates embryonic transcriptional programs that enable dedifferentiation, lineage switching, and adaptive tumor evolution. This reprogramming involves the suppression of luminal differentiation markers, thereby facilitating the loss of AR dependency and promoting a basal-like or NE phenotype.^51,54^ Targeting key regulators, such as Sox2, or the pathways activated by the combined loss of *RB1*, *TP53* and *Pten*, may offer new therapeutic strategies to overcome resistance and prevent tumor evolution in advanced prostate cancer.

NE transformation exhibits distinct subtype mechanisms in NEPC and SCLC, each driven by unique combinations of genetic and epigenetic alterations. In NEPC, *MYCN* overexpression and *RB1* loss can occur independently or co-occur in patients and have been identified as key drivers of NEPC. To model this, *Pb-Cre4*^*+/–*^*;Pten*^*f/f*^*;Rb1*^*f/f*^*;LSL-MYCN*^*+/+*^ (*PRN*) mice were generated, leading to MYCN overexpression in prostate epithelium alongside *Pten* and *Rb1* deletion. Analysis of these models revealed that MYCN and RB1 cooperate to foster the NE differentiation into distinct lineages, including ASCL1^+^ and POU2F3^+^ subtypes. The ASCL1^+^ subtype follows canonical neuronal differentiation pathways, marked by the expression of neuronal genes associated with synaptic functions. In contrast, the POU2F3^+^ subtype represents a rare tuft cell-like lineage, characterized by MYCN-mediated chromatin reprogramming and a mutual exclusivity with ASCL1 expression. These divergent subtypes highlight the role of MYCN as a master regulator of chromatin dynamics, enabling NEPC cells to adopt diverse NE identities.^145^ In SCLC, NE transformation mechanisms are similarly complex, with tumors often displaying varying expression levels of lineage-defining markers such as ASCL1, NEUROD1, and POU2F3.^146^ A distinct subtype with YAP1 expression has been identified, demonstrating low levels of canonical NE markers. Using a series of lineage tracing GEMMs, including *Cgrp*^*CreER*^*;Trp53*^*f/f*^*;Rb1*^*f/f*^*;Pten*^*f/f*^ (TKO (triple knockout), *Cgrp*^*CreER*^;TKO;*Rosa26*^*mTmG*^, and inducible YAP overexpression models (*Cgrp*^*CreER*^;TKO;TetOn-*YAPS127A* and *Cgrp*^*CreER*^;TKO;TetOn-*YAP5SA*), in combination with *Rbpj*^*f/f*^ to modulate Notch signaling, these studies demonstrated that YAP activation promotes a dynamic transition from NE to non-NE states. Mechanistically, YAP interacts with transcriptional regulators such as REST and Notch2, leading to the suppression of neuronal genes like ASCL1 and the induction of non-NE markers, including HES1 and CC10. This NE-to-non-NE plasticity highlights YAP’s role as a key modulator of lineage state transitions, enabling tumor cells to adapt to therapeutic and microenvironmental pressures.^147^

NE transformation is not solely driven by single factors such as *ASCL1* expression or *RB1* loss but rather involves a complex interplay of genetic, transcriptional, and epigenetic regulators. For instance, while *ASCL1* expression and *RB1* loss are necessary, they are insufficient to fully convert AR-positive or CRPC cells into NE phenotypes. Research by Cejas and colleagues identified SRRM4 as a novel and critical driver of NE transformation in prostate cancer. SRRM4 reprograms alternative splicing to activate NE-specific gene expression by modifying the function of REST, a transcriptional repressor of neuronal genes. SRRM4 induces the splicing of REST into a truncated isoform, REST4, which lacks functional repressor domains. This splicing event derepresses neuronal and NE-specific genes, including *ASCL1*, *INSM1* and *SYP*, thereby facilitating the transition to an NE phenotype.^148^ This discovery underscores the importance of alternative splicing as a regulatory mechanism in NE plasticity.

GEMMs of SCLC have been instrumental in replicating the core genetic alterations observed in human tumors, such as *TP53* and *RB1* loss and *MYC* amplification. These models have provided crucial insights into subtype heterogeneity, tumor progression, and therapy resistance. Additionally, advances in CRISPR/Cas9-based somatic editing and immunocompetent models have enabled the study of tumor–immune interactions, offering new opportunities to dissect therapeutic vulnerabilities in NE cancers. These state-of-the-art models allow for the exploration of how NE plasticity influences immune evasion, microenvironmental adaptation, and lineage-specific dependencies.^149^

### Conceptual models of plasticity leading to histological transformation

**Fig. 5:**
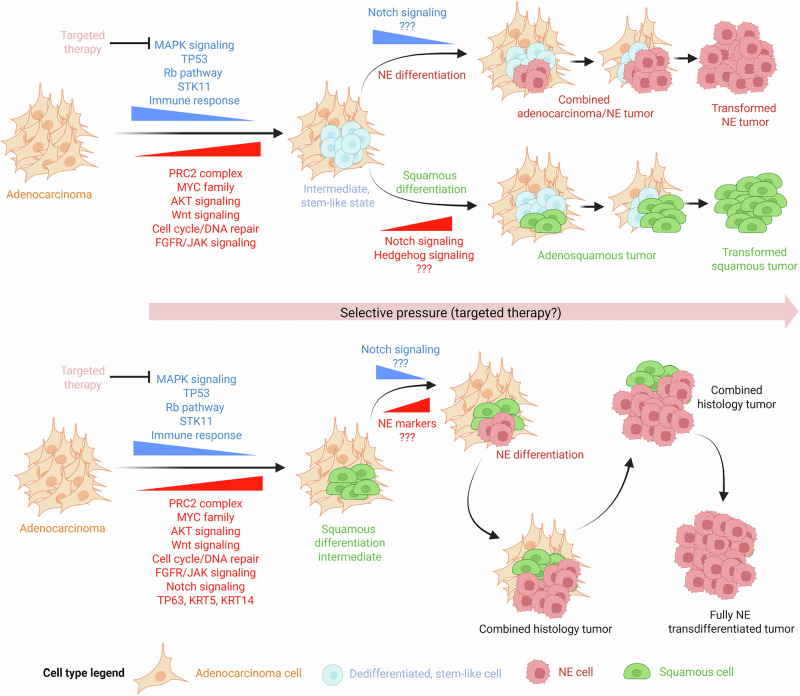
Recent evidence supporting alternative models of histological transformation. Models of transformation during selective pressure-driven tumor evolution (arrows), such as targeted anti-tumor therapy. Two models are depicted, histological transformation through a dedifferentiated intermediate stem-like state (top) or through a squamous-like intermediate state (bottom). Cell type legend can be found at the bottom of the figure. Question marks account for yet undescribed additional molecular alterations potentially driving transition among different cell states. Figure generated with BioRender.com.

Recent molecular analyses in clinical specimens and preclinical models have identified dysregulated pathways during NE and squamous transformation.^6,38^ Surprisingly, several oncogenic pathways were commonly upregulated in both NE- and squamous-transforming clinical specimens, including pathways related to cell cycle and DNA repair, the PRC2 epigenetic remodeling complex, AKT and Wnt signaling, as well as MYC targets, the latter induced by MYC or MYCN.^6,38,68,145^ Similarly, several pathways related to the anti-tumor immune response were downregulated in both NE and squamous transformation, suggestive of a strong anti-tumor immune suppression occurring during histological transformation.^6,38^ Also, as previously mentioned, *STK11* inactivation has been reported as a promoter of the squamous and large cell carcinoma phenotypes in GEMMs,^100^ and in potential relationship with the latter, *STK11* inactivation is also hallmark of a subset of LCNECs enriched for mutations of LUAD drivers such as *KRAS,*^45,106^ which could potentially be cases of NE transformation. These observations associate *STK11* inactivation with plasticity and suggest a role for this molecular event as an enabler of plasticity, which could potentially lead to different histological outcomes. Additionally, there is evidence of oncogenic pathways exclusively dysregulated in LUSC transformation, like Hedgehog signaling, and even pathways showing opposite dysregulation depending on the histological fate of transformation, such as Notch signaling, suppressed and induced in NE^116^ and LUSC^38^ transformation, respectively. The apparent convergence and divergence of molecular events in NE and squamous transformation, as above explained, suggest a model of transformation where these convergent molecular alterations may lead to a potential intermediate dedifferentiated state, common for histological transformation independently of the histological outcome. Indeed, this observation is supported by the fact that many of these have been extensively associated to stemness.^110,150–152^ After this intermediate state has been reached, the occurrence of additional molecular events, potentially those found to occur divergently in LUSC and NE transformation, may ultimately decide the histological outcome (Fig. 5, top).

On the other hand, there are data supporting an alternative model where the squamous/basal-like histology may represent the intermediate state of adenocarcinoma-to-NE transition. Even if most combined histology tumors show only two different histological components, the occurrence of tumors exhibiting multiple histologic subtypes during transformation has been documented. Examples of this include baseline prostate adenocarcinomas transdifferentiating into a combined squamous/NE/sarcomatoid tumor,^153^ or lung tumors with adenocarcinoma, squamous, and NE histological components.^154^ One potential explanation for these could be the divergent evolution of different adenocarcinoma clones, each transdifferentiating into a different histological subtype, but that seems unlikely. Analogous to what was observed in clinical specimens, preclinical evidence in GEMMs of histological transdifferentiation indicate the co-existence of different histologies. In the NPp53 model, with combined inactivation of *Trp53* and *Pten* in the prostate epithelium, treatment with the androgen blocker abiraterone induced histological transdifferentiation leading to tumors with areas of squamous/basal, sarcomatoid, small cell-like NE, and other non-adenocarcinoma phenotypes.^155^ In two other GEMMs, one with inactivation of *Rb1* and *Pten*, simultaneously with *MYCN* transgene overexpression,^145^ and the other with prostate-specific inactivation of three tumor suppressors *Trp53*, *Rb1* and *Pten*,^65^ we observe tumors exhibiting combined histology with AR-positive adenocarcinoma, undifferentiated areas with intermediate adenocarcinoma/NE phenotype, and foci exhibiting squamous differentiation. Importantly, analyses of these models suggest that early steps of plasticity may lead towards a basal-like phenotype characterized by the expression of squamous markers, such as Krt5, Krt14 or Trp63. In line with this, transcriptomic upregulation of basal markers is observed in prostate adenocarcinoma cell lines after the sole inactivation of *TP53* and *RB1*.^51^ Similar results were obtained in a preclinical model leveraging human prostate epithelial cells with concurrent inactivation of *TP53*/*RB1*, as well as overexpression of MYC, BCL2 and a constitutively active AKT isoform.^68^ Engraftment in vivo of such cells leads to the formation of tumors again exhibiting multiple histologies such as adenocarcinoma, squamous, small cell carcinomas, and mixed phenotypes. Temporal analyses of marker expression in this model revealed early-stage upregulation of basal/squamous cell markers such as TP63, and late-stage upregulation of NE markers such as SYP or NCAM1.^156^ These results are suggestive of a potential model of transformation where adenocarcinomas, or a subset of them, may transition through a squamous phenotype towards their final NE state (Fig. 5, bottom).

**Fig. 6:**
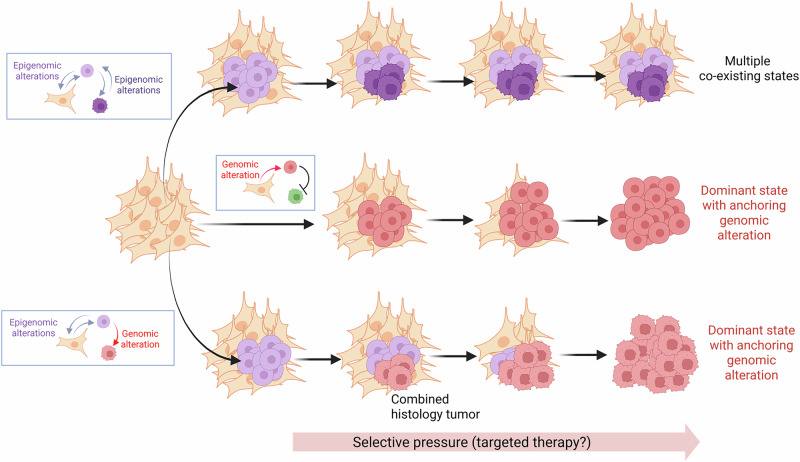
Model of histological transformation where dysregulation of plasticity-related pathways may occur through reversible epigenetic changes or through irreversible genomic events, leading to differential evolution of plastic tumors. Cells with purple color tones represent cells which underwent reversible epigenomic changes, while red cells represent cells where irreversible genomic alterations were acquired. The green cell represents a cell state to which a red cell cannot transition, as it has acquired an irreversible genomic alteration that anchors the cell in the green state. Figure generated with BioRender.com.

Although it is thought that most molecular changes occurring during histological transformation are governed by epigenomic events,^6,38,157^ tumors undergoing this process frequently exhibit genomic alterations inducing dysregulation of drivers/promoters of plasticity. For example, in NE transformation, ~90% of tumors exhibit copy number loss or inactivating mutations in *RB1* vs only 10% showing wild-type *RB1* with protein downregulation, and transforming tumors often exhibit activating mutations/amplifications in gene members of the AKT pathway.^6^ If histological transformation is the result of the convergent dysregulation of promoter/driver genes or pathways, when such dysregulation is driven by epigenomic events, it might be reversible. Thus, cells might be free to transition between different epigenomic states (Fig. 6, top). However, when the pathway dysregulation occurs as a consequence of a genomic event, which is less likely to be reversible, this may anchor the cells in a particular state (Fig. 6, middle). Both molecular scenarios may coexist, with the occurrence of (1) co-existence and interconversion between histological subtypes (reversible, changing epigenomic states) as part of an equilibrium, that may or may not be temporary; and of (2) genomic alterations anchoring the cells in a given state, such as squamous or undifferentiated phenotypes, thus preventing transition to NE carcinoma, and resulting in a squamous transformation or undifferentiated tumor with loss of adenocarcinoma features.

### Therapeutic targets to constrain plasticity and histological transformation

Two key considerations inform therapeutic approaches to combat tumor plasticity, and we choose to view these as potential questions for consideration, (1) are pre-existing, therapy-resistant subclones with transcriptomic and/or morphological features of an alternative histology present prior to therapeutic intervention and therefore likely to be selected for over time with adequate pressure; or, (2) does therapeutic pressure directly convert some part of the measurable residual disease into a therapy-refractory disease, now presenting with differing histologic features?

While both scenarios are possible, their differences may impact the design of optimal therapeutic approaches, where different drugs may be combined or staggered, depending on variables including the kinetics of resistance and additive toxicities of each approach. If the goal of any treatment strategy is to prolong healthy years of life for the patient, strategies prioritizing the temporal control of disease over maximal depth of response may be appropriate.^158^ However, during consolidation of primary lesions, several key points about control of residual disease are important to stress. Using EGFR-driven LUAD as an example, despite the impressive responses and clear clinical benefit to the TKI osimertinib, now widely used and FDA-approved in the first-line setting, the median best percentage change in target-lesion size in approval trials was ~54%, thus leaving substantial residual disease to address.^159^ Next, following any large debulking event, the residual disease is different, now immersed in a fibrotic, necrotic, and often hypoxic TME that may or may not have been present prior to starting therapy.^160,161^ These differences may impact the exposure, and thus efficacy, for any therapy introduced following an objective response.

Therefore, targeting mechanisms responsible for histological transformation may be broadly grouped into several categories, including (1) epigenetic mechanisms thought to be responsible for mediating the transitions between adenocarcinoma and NE tumor types; (2) conduction of replication stress associated with a change in oncogenic driver programs; (3) upstream signaling mechanisms responsible for the survival and immune evasion of the residual disease; and (4) molecular features of NE or adeno-squamous tumor types that are uniquely present following histological transformation.

### Exploiting epigenetic plasticity as a therapeutic approach for NE tumors

Loss of the *RB1* tumor suppressor plays a key role in unveiling epigenetic heterogeneity in advanced cancers.^162–166^ While the direct transcriptional activity derived from *RB1* inactivation may not be pharmacologically targetable yet, *RB1* inactivation leads to increase of dependency on enzymatic targets susceptible for pharmacological inhibition. These include critical regulators of cancer progression and/or NE transition — namely LSD1^167^ and EZH2.^168,169^

Studies in CRPC models have demonstrated that LSD1 inhibition may act through disruption of enhancer networks that are partially facilitated by MYC and BET protein interactions.^167,170^ More has been demonstrated in the context of SCLC, where deletion of *Kdm1a* has been shown to suppress de novo SCLC tumorigenesis, partly through the upregulation of *Rest*, thus directly repressing *Ascl1* target gene activity.^171^ Additional preclinical studies have demonstrated that exceptional responses to LSD1 inhibitors (including ORY-1001 and GSK2879552) may occur through the activation of Notch signaling, leading to direct or indirect suppression of Ascl1.^172,173^ Additional follow-up studies have found that genes involved in MHC-1 expression are upregulated following LSD1 inhibition, thereby potentiating the activity of immune checkpoint blockade.^174,175^ This is a critical finding, as patients with SCLC experiencing the greatest benefit from immune checkpoint blockade are those with the weakest mRNA expression of *LSD1* and *EZH2* or the greatest expression of an antigen presentation machinery pathway, specifically *HLA-A*, *HLA-B*, *HLA-C*, *B2M*, *TAP1*, and *TAP2*.^176^ Whether these findings are extendable to NEPC remains to be determined.

Chemical inhibition of EZH2 has been demonstrated to suppress the emergence of anti-androgen resistance — both EZH2 and SOX2 have been described as critical “re-programming” factors in NE transformation.^51,54^ Indeed, there may be substantial overlap in how chemical inhibition of the enzymatic activity of EZH2 or LSD1 may impact epigenetically silenced antigen presentation and oncogenic driver pathway activity. Ongoing trials will help directly address whether EZH2 inhibitors can be safely combined with AR-targeted therapy and delay the emergence of CRPC and/or NEPC (NCT04179864, NCT03460977). Collectively, these studies highlight the importance of addressing two inter-related issues underscoring immune evasion in NE transformation — antigen presentation and the added reinvigoration of immune effector function. On the lung side, EZH2 pharmacological inhibition showed no efficacy in combination with osimertinib in an NE-transformed, *EGFR-*mutant PDX model,^6^ although the efficacy of this therapeutic approach at preventing transformation is yet to be investigated in LUAD models undergoing NE transformation. In an *EGFR-*mutant PDX model of LUSC transformation, exhibiting high sensitivity to osimertinib, the combination of osimertinib with an EZH2 inhibitor led to suppression of LUSC relapse in vivo.^38^ This combination was also efficacious at reverting resistance to osimertinib in this same PDX model, after LUSC relapse on osimertinib. Such results suggest that EZH2 inhibition may be a promising therapeutic strategy for tumors undergoing LUSC transformation.

Clinical trials combining either LSD1 or EZH2 chemical inhibitors with SCLC-centric cytotoxic chemotherapy may have seemed logical based on preclinical mechanism;^177^ however, two points of caution should be raised based on current trial results — (1) toxicity may be limiting, and thus preclude the combination of certain cytotoxic chemotherapies with EZH2 catalytic inhibitors,^178^ and (2) if LSD1 inhibition does suppress an Ascl1-dependent transcriptional program, then this may also suppress the chemotherapy-sensitive NE fraction, limiting activity.

Another epigenetic effector sustaining the NE phenotype in both prostate and lung cancer settings may represent an alternative therapeutic option — the SWI/SNF chromatin remodeling complex, whose catalytic subunit, SMARCA4, is highly expressed in NE tumors.^179,180^ SWI/SNF complexes interact with different lineage-specific factors in NEPC compared to prostate adenocarcinoma.^179^ In the lung setting, SMARCA4 genetic or pharmacological inhibition leads to the loss of NE features and transition towards a non-NE, adenocarcinoma-like state with induction and overactivation of HER family receptor tyrosine kinases^180^ and MAPK signaling, known to be incompatible with the NE phenotype.^181^ Combined treatment with a SMARCA4 small-molecule inhibitor and the HER receptor inhibitor afatinib exhibited high efficacy in an array of SCLC PDXs. The availability and further development of potent and selective SMARCA4 inhibitors and degraders offers a promising therapeutic strategy for NE tumors.

### Targeting replication stress

A hallmark of solid tumor types with the losses of tumor suppressors *RB1* and/or *TP53* are features of high replication stress, genomic instability, mitotic/proliferative indices, and general transient sensitivity to DNA damaging chemotherapy.^62,182–184^ The list of therapeutic targets exploiting vulnerabilities in replication stress is ever-expanding, but in general, can be grouped into agents that impact DNA fidelity directly (i.e., GC-groove binding agent lurbinectedin^185^) or indirectly (i.e., DNA damage signaling and repair machinery). Most substantially advanced from the bench to the patient have been the translation of PARP and ATM/ATR inhibitor therapies.^186–190^ For example, a recent trial combining the ATR inhibitor barzosertib with topotecan demonstrated a significant extension in overall survival in relapsed SCLC but fell short in achieving the primary endpoint of extending progression-free survival.^191^ While such agents have demonstrated clinical activity in relapsed SCLC, CRPC, and NEPC, the search for biomarkers to help identify patients most likely to benefit as well as mechanistic explanations for exceptional responders is an on-going pursuit. It is important to emphasize that the overall challenge remains in identifying therapies with durable responses, as most patients have been found to progress on study in less than one year. Such limited duration of efficacy falls short of the observed benefits of upfront targeted therapies, like ADT and EGFR TKIs.

In pursuit of identifying mechanisms responsible for cross or “pan” resistance to agents that exploit replication stress, a recent study modeling acquired resistance using patient-derived models of SCLC found that *MYC* family paralog amplification via ecDNA drives acquired cross-resistance by amplifying replication stress response pathways and altering transcriptional landscapes.^70,192^ Recent evidence in mouse models from our groups suggests that the pulmonary NE cell can be transformed by the *Myc* oncogene alone, thus representing a fundamental oncogenic driver event. Furthermore, MYC may have a critical role in driving histological transformation of lung cancers to either a NE or squamous fate.^6,38,142^ Whether or not NE cells within other tissue, namely the prostate, are directly transformed by any Myc family alone remains to be determined; however, several key studies have clearly demonstrated that Myc family members including cMyc and nMyc are capable of cooperating with truncal, inactivating events in *Rb1*, *Trp53*, and/or *Pten*, to produce aggressive, metastatic NEPC.^74,193–195^ Pharmacological inhibition of MYC family members has proven challenging.^196^ The original studies demonstrating potent, direct MYC inhibition made use of a peptide (Omomyc) that was rationally designed to compete with the Myc–Max interface, acting as a dominant-negative Myc protein.^197^ This concept has been improved over the last two decades and has recently entered clinical testing in solid tumors.^198,199^ Additional preclinical strategies to target the MYC–MAX interface have also been recently introduced and hold promise for preclinical translation.^200^ Indeed, prior data in SCLC models suggested that Omomyc had potent in vitro activity.^201,202^ On the other hand, our group has recently identified a therapeutic target, the cell division cycle 7 kinase (CDC7), whose inhibition induces indirect MYC inactivation by its proteasomal degradation, thus hindering NE transformation and significantly extending response to targeted therapy in both lung and prostate tumor models.^60^ Perhaps these results are not all that surprising, as SCLC is the only solid tumor found to lose the MYC-binding effector MAX as a mechanism of tumor progression, further highlighting a role for MYC in SCLC tumorigenesis.^203,204^

Considering that NE cancers are bi-modally distinct from adenocarcinomas based on the expression and activity of YAP/TAZ,^205^ histological transformations can be understood as complex events involving both genetic and epigenetic remodeling over large timescales. Several recent studies have focused on the intermediate phases of tumorigenesis leading up to histological transformation. In prostate cancer models of acquired resistance to AR-targeted therapies, a critical role for JAK/STAT signaling has been identified in promoting an inflamed, stem-like precursor to NEPC.^63,65^ This same stem-like intermediate has been observed in other studies where in vivo systems were used to chart the transformation of normal luminal and/or basal precursor cell types into NE tumor types.^68,156^ However, it remains unclear when and how an inflamed or stem-like state may help evade key immune checkpoints in tumor control, leaving this as an area of active investigation. Interestingly, JAK/STAT signaling may also play a role in promoting the adeno-to-squamous transition observed in some LUADs following treatment with targeted therapies, including recently approved KRAS inhibitors.^4,104,105,206^ Encouragingly, recent activity combining the JAK/STAT inhibitor itacitinib with immune checkpoint blockade has shown promise in advanced LUAD.^207^ These findings suggest a broader role for JAK/STAT signaling in mediating lineage plasticity across different cancer types. Collectively, the inhibition of JAK/STAT signaling raises the possibility of trapping an intermediate state during histological transformation that is both inflamed and expressing MHC-1 prior to transformation to an NE state. Understanding the temporal relationship between an early inflamed TME of an adenocarcinoma, the introduction of immune checkpoint blockade to exploit this inflammation, and the eventual acquisition of a cold TME of the NE state are active areas of research. Collectively, such an approach may have the potential to limit the full transition to NE phenotypes while simultaneously enhancing immune-mediated tumor control.

Along similar lines of reasoning, targeting of the PI3K/AKT signaling pathways has also been proposed to address the survival of the residual disease and directly target a common genetic alteration found in advanced prostate cancer.^208–210^ Several smaller trials in CRPC patients have explored the combination of either mTOR, PI3K, or AKT inhibitors with chemotherapy or ADT and have not demonstrated benefit, some with toxicities that limited the dosing intervals.^211–215^ Mutations in the PI3K/AKT/mTOR pathway are frequent in NE transformation,^6,216^ and overactivation of the pathway has been associated with NE transformation in the lung and prostate^68,155^ and even with squamous transformation.^38^ The fact that this pathway may have a promoting role in either squamous or NE transformation, and that its dysregulation occurs in adenocarcinomas with no NE features,^217^ is suggestive of a potential role for this pathway to contribute to the previously mentioned initial, stem-like state prior to histological transition. In the lung setting, PI3K/AKT/mTOR pharmacological targeting has been tested in preclinical models of transformation. In the NE transformation setting, the pathway inhibitor samotolisib was combined with osimertinib in an *EGFR-*mutant PDX model of NE transformation.^38^ Even if the combination slowed tumor growth more than either monotherapy alone in this model, the response observed was modest. Importantly, this particular PDX model exhibited baseline combined histology with both LUAD and NE components, with predominant NE histology, and was not responsive to osimertinib. This observation suggests that such a therapeutic approach might not be effective after transformation has occurred and opens the question of whether this combination therapy may hinder plasticity if administered before transformation. In line with this, analyses of tumors collected after treatment in this model revealed that samotolisib avoided the complete disappearance of the LUAD component observed after osimertinib monotherapy, suggesting that PI3K/AKT/mTOR inhibition may be a promising strategy in tumors at high risk of NE transformation. Targeting of PI3K/AKT/mTOR has also been tested in an *EGFR-*mutant PDX model of LUSC transformation,^38^ which exhibited limited sensitivity to osimertinib. In this model, samotolisib resensitized tumors to osimertinib, suggesting that AKT inhibition may be a promising strategy for LUSC-transformed tumors. However, the efficacy of such inhibitors in preventing LUSC transformation is yet to be studied. Unfortunately, the toxicity of current PI3K/AKT/mTOR inhibitors, which would be potentiated if combined with targeted therapy, may be a limitation to bring these drugs into the clinic successfully. Although prior efforts to inhibit this signaling axis have been disappointing, this does not preclude the target as relevant, where perhaps alternative approaches (i.e., AKT degraders^218^) leading to reduced toxicity will have beneficial activity.

### NE-specific vulnerabilities

Historically, one of the best examples of “good target, wrong approach” is the DLL3-targeted therapies. Originally identified as elevated in high-grade NE cancers — including SCLC, LCNEC, and NEPC — DLL3 functions as a Notch inhibitory ligand and was considered “targetable” due to its unique expression in tumors relative to normal tissues.^30,219–221^ However, later-phase translational efforts utilizing antibody–drug conjugates (ADCs) encountered significant setbacks, as poor stability between the monomethyl auristatin E warhead and the DLL3-targeting antibody precluded further development of this approach.^222^ Despite these early challenges, the field has since evolved, encompassing new approaches such as traditional ADCs, BiTEs, radioimmune conjugates, and others, all focused on DLL3.^223^ In May 2024, tarlatamab was FDA-approved for extensive-stage SCLC with disease progression after platinum-based chemotherapy.^32^ With preclinical evidence suggesting that DLL3 is similarly expressed in NEPC and that targeting DLL3 has therapeutic potential,^224,225^ several ongoing clinical trials aim to broaden the indications for tarlatamab and other DLL3-related therapies (NCT04702737, NCT05652686, NCT05882058).

Recently, our group identified exportin 1, a nuclear exporter, as a promising therapeutic target in NE tumors, including SCLC and NEPC.^226,227^ Exportin 1 was found to be upregulated during the very early stages of NE transformation, immediately following *TP53* and *RB1* inactivation. Inhibition of exportin 1 with selinexor significantly extended chemotherapy response in various PDXs of SCLC and NEPC. Selinexor also prevented NE transformation in multiple in vivo models of prostate and lung cancers, greatly enhancing the efficacy of targeted therapies in both settings. Mechanistic studies revealed that exportin 1 inhibition downregulated SOX2.^227^ These findings, coupled with the clinical availability of selinexor, which is FDA-approved for treatment-refractory hematological malignancies, position exportin 1 as a compelling target to prevent plasticity in these aggressive tumors.

Additional targets implicated in NE transformation have emerged from analyses of de novo SCLC and histologically transformed samples, including B7-H3/CD276, B7-H6, SEZ6, TROP-2, CEACAM5, and others.^228–234^ Therapeutic strategies targeting these molecules are diverse, including ADCs, BiTEs, chimeric antigen receptor T cell therapies, and other modalities. However, no clear “winning” approach has yet been identified, fueling continued preclinical development. Furthermore, as multiple NE-targeted therapies gain FDA approval, questions regarding the timing and sequence of these treatments remain unanswered. It is not yet clear how recurrent disease will present (e.g., DLL3 positive or negative, NE or not) or whether specific subtypes of NE tumors, such as ASCL1^+^ vs NEUROD1^+^, will preferentially benefit from distinct therapeutic strategies.^235^

## Open questions

Despite progress during the last five years to identify promoters, effectors, and therapies targeting histological transformation, several open questions remain to be addressed. Most urgent in our opinion, is the identification of molecular biomarkers predictive of squamous transformation. Whether certain genetic or epigenetic events lock transdifferentiation of adenocarcinoma into a squamous-like state over an NE one is not well defined and remain areas of active investigation. Accrual of large numbers of genetically annotated samples from patients on institution-specific trials continues to present logistical challenges. Moreover, to date, molecular characterizations of squamous transformation have been limited, with no clear emergent molecular predictors of high risk. In addition, the scarcity of human and mouse preclinical models of squamous transformation represents an important challenge in the validation of potential therapeutic targets to prevent or treat these tumors.

Another open question relates to the molecular differences between de novo and transformed tumors, and whether these may respond differentially to therapy. Molecular analyses of clinical specimens undergoing NE or squamous transformation have revealed that transformed tumors may retain broad-scale molecular features of their previous adenocarcinoma state, and may exhibit greater heterogeneity,^5,6,38^ potentially making them a different entity from de novo occurring NE and squamous tumors. Particularly in the transformed SCLC setting, it has been suggested that transformed tumors may respond worse to chemotherapy as compared to de novo tumors, with a slightly inferior progression-free survival of 3.4 months vs 5.5 months.^236,237^ However, these results are yet to be confirmed. Importantly, in the *EGFR-*mutant LUAD setting, we know that tumors lose expression of EGFR upon NE transformation. However, we still do not know whether that is also the case for tumors undergoing LUSC transformation, or whether in adenocarcinomas undergoing histological transformation with drivers different than EGFR, the adenocarcinoma driver gets downregulated as well.^238,239^ Understanding the molecular biology of transformed tumors will be key to identifying effective therapeutic targets.

The influence of the TME in the process of histological transformation also remains undefined. Even if slight upregulation of NE or squamous markers can be measured in vitro in adenocarcinoma cell lines and organoid models with the appropriate molecular modifications,^38,51^ major transformation features are only observed in vivo.^65,240^ This observation suggests that the TME may have a role in histological transformation. Consistent with this hypothesis, clinical specimens undergoing NE or squamous transformation exhibit a strong downregulation of a few pathways related to immune response,^6,38^ which may indicate the requirement of a strong suppression of anti-tumor immune response for transformation to occur. As epigenetic reprogramming to a stem-like state is an immunogenic process,^241–243^ this immune suppression may be exerted by tumor cells to protect themselves from the immune system during transformation.

One interesting and potentially key aspect of histological transformation that remains unknown, briefly covered in the present review, is whether there is a strict directionality in transformation. In other words, can histological transformation be reverted? On the NE transformation side, reports indicate that MAPK induction may be incompatible with NE SCLC, where it induces cell cycle arrest and senescence.^181^ These results indicate that EGFR (or receptor tyrosine kinase) re-expression, which would lead to MAPK signaling, may be challenging in SCLC tumors. Interestingly, recent analyses of clinical specimens and GEMMs have described the existence of plasticity in SCLC. Even if SCLC tumors exhibit homogeneous morphology, different transcriptomic subtypes have been identified with different levels of NE phenotype.^146^ These subtypes are not static, but plastic, in a way that NE SCLC tumors can transition to a non-NE state with an EMT phenotype and activation of Notch signaling.^119^ Indeed, as mentioned above, inhibition of the activity of enzymes such as EZH2 or LSD1, thought to be partially responsible for the epigenomic remodeling observed following histological transformation, may induce an NE to non-NE transition.^75,174,175^ These results indicate that the reversal of the NE phenotype may be possible, but whether additional molecular alterations lead to reprogramming (or “resetting”) all the way back to the original adenocarcinoma phenotype remains unknown.

Finally, a related open question is whether transformation may occur in both directions, with an equilibrium between the adenocarcinoma and the alternative histologic subtypes present in the same tumor. The occurrence of combined histology tumors, particularly adeno-squamous tumors which are relatively frequent representing 2%–4% of lung tumors,^244^ could indicate that (1) tumors were collected at an intermediate state of transformation, before the initial state (adenocarcinoma?) gets overgrown by the transformed cells; or (2) that there might be an equilibrium of interconversion between cells from both histologic subtypes induced by a selective advantage derived from both cell types being present in a given tumor, such as a potential cellular interaction between both components supporting oncogenicity and progression, as has been reported in other settings.^121,122^

**Fig. 7:**
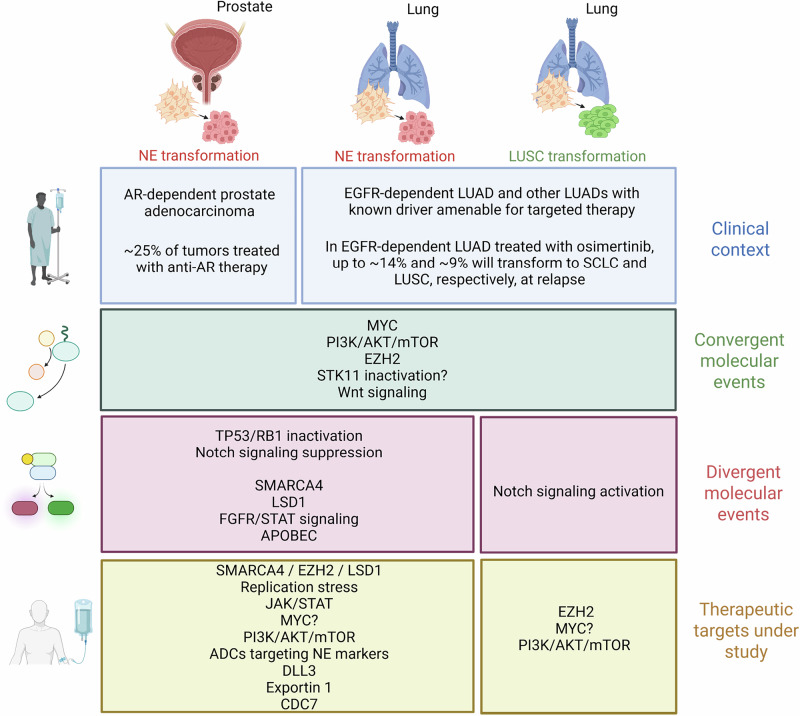
Summary of clinical context, molecular alterations and therapeutic approaches being studied for the different most studied histological transformation settings in the lung and prostate. This includes NE transformation in prostate cancer, and NE (SCLC) and LUSC transformation in LUAD. AR, androgen receptor. Figure generated with BioRender.com.

All data reviewed in the present manuscript highlight the challenges that plasticity poses to the clinical management of tumors able to transition to different states via dysregulation of specific oncogenic pathways (Fig. 7). Both the cell of origin and the driver oncogenic signaling derived from the tumor at a given time may induce contextual molecular constraints in terms of the histological phenotypes that a given tumor may display. However, the inhibition of that particular driver, pharmacologically or otherwise, together with the convergence of the right molecular alterations may overcome those constraints. In this sense, histological subtyping, even if of clinical relevance, may be a rough categorization of what could potentially be a spectrum of phenotypes defined by the activation of a given combination of oncogenic pathways. Indeed, in spite of efforts to design molecular tests to accurately predict tumor histology, including genomic and epigenomic approaches,^28,245,246^ currently histological subtyping in the clinic relies on microscopic morphological criteria and differential expression of differentiation immunohistochemistry markers. Intermediate or transitioning phenotypes may certainly pose a challenge for the development of such molecular tests. An “all-plastic” model (Fig. 5) has been proposed, positing that tumors may be able to transition from one state to another in response to certain selective pressures (treatments, hypoxia, TME, etc.), unless a state-defining oncogenic pathway occurs in a genomic, irreversible manner (as described in Fig. 6). Additionally, the occurrence of tumors with combined histology, intermediate phenotypes, or undifferentiated/dedifferentiated phenotypes, mentioned in the present review, would be explained by this plastic spectrum model. Nonetheless, additional confirmatory work will be required to support or refute such a model, where the use of cutting-edge single-cell and spatial techniques, as well as the development of potent, reliable lineage tracing methods will be key to studying directionality of transformation and fully characterizing intermediate/admixed histological phenotypes.

## Conclusions

Recent multi-omic analysis of clinical specimens and single-cell profiling of preclinical models of plasticity have identified oncogenic pathways dysregulated during histological transformation. Some of these may be promoters of plasticity and some drivers of transdifferentiation towards a specific state (i.e., manifesting as a tumor “histology”). Importantly, intermediate states of transformation have been identified that may be of particular therapeutic relevance — specifically an inflammatory intermediate bearing hallmark of EMT. Early promoters of plasticity driving such states may be key therapeutic targets to prevent or delay histological transformation in patients at high risk. Upcoming studies leveraging state-of-the-art high-resolution, spatial and lineage tracing technologies, will be key to further characterizing the directionality, signaling timing of histological transformation and its potential reversibility, as well as the role of the TME in this plasticity phenomenon.
